# “Becoming a Medical Cannabis User” Revisited: Understanding the Role of Low-Threshold Access Points in British Columbia, Canada

**DOI:** 10.1177/00914509241312872

**Published:** 2025-01-22

**Authors:** Marilou Gagnon, Heather Hobbs

**Affiliations:** 1 School of Nursing, 8205 University of Victoria, Victoria, BC, Canada; 2 Canadian Institute for Substance Use Research, 8205 University of Victoria, Victoria, BC, Canada

**Keywords:** access, cannabis, cannabis clubs, dispensaries, legalization, low-threshold

## Abstract

The work of sociologist Howard Becker has been used extensively to study and theorize the experience of using cannabis for recreational purposes and more recently, the experience of using cannabis for medical purposes. Building on recent research that uses Becker's work to study and theorize the *process of becoming a medical cannabis user*, we extracted and analyzed interview data from a larger qualitative case study of low-threshold access points (i.e., medical dispensaries and cannabis clubs) in British Columbia (Canada). The majority of participants were 50 years or older, had an annual income of less than $30,000, listed disability assistance as their primary source of income, and were renting a room or an apartment. Educational level was distributed across the sample and gender representation was close to balanced with seven cisgender women and five cisgender men. All of the participants identified as White (European descent). In terms of cannabis consumption, the majority of participants reported using daily and indicated a preference for ingesting cannabis, followed by smoking and applying topically. Our theory-informed analysis suggests that becoming a medical cannabis user is a process that requires low-threshold access to community, medicine, and space—three domains across which learning unfolds, in relationships (with staff and peers) and in response to health and social needs. Our analysis also points to three categories of learning: learning about medical cannabis, learning to medicate, and learning to substitute. Overall, our findings indicate that learning across all three categories *and* low-threshold access to community, medicine, and space go hand in hand in the process of becoming a medical cannabis user—thus generating new theoretical insights and empirical avenues. The findings also raise important questions about the closure of low-threshold access points in British Columbia and the impact of cannabis legalization more generally.

The work of Howard Becker (1953, 1955) has been used extensively to study and theorize the experience of using cannabis for recreational purposes and more recently, the experience of using cannabis for medical purposes. Becker has been credited for shifting the focus away from the individual using cannabis, as someone who possesses individual traits that explain their cannabis use, toward the behavior itself, which he argued is learned, managed, and integrated in social contexts and through social relations (Hathaway, 1997). Becoming a (recreational) cannabis user, he explained, is a social process that requires a person to learn how to smoke cannabis properly, understand what “being high” feels like, and define this experience as a pleasurable one (Becker, 1953). According to Becker (1953), this process of becoming necessarily requires *and* involves some form of social learning and interacting with experienced cannabis users. This is particularly true for beginners, the first career stage identified by Becker, but as a person moves to become an occasional cannabis user or a regular cannabis user, the second and third career stages, the social dimensions of cannabis use remain important and contribute to normalizing the experience against broader social controls that are intended to prohibit and discourage cannabis use (Hathaway, 1997). In his original work, Becker (1955) highlighted three social controls, namely supply (i.e., prohibition aimed at limiting cannabis use and access to cannabis), secrecy (i.e., fear of disclosure and social consequences), and morality (i.e., social norms contributing to stigma). However, as scholars have noted since, these social controls should be revisited to reflect changes in social norms, broader normalizing of cannabis use, and legal reforms (e.g., decriminalization and legalization of medical and/or recreational cannabis) (Hathaway, 1997; Hathaway et al., 2011; Järvinen & Ravn, 2014). Other scholars, such as Hathaway (1997) and Hallstone (2002), have also identified the need to rethink the process of becoming in light of social changes, such as access to information online, which may decrease the importance of learning from and interacting with others, and emerging evidence pointing to the individual nature of leisure and pleasure as well as the intuitive aspect of cannabis when used recreationally. Finally, some scholars have recommended additions and revisions to the career stages developed by Becker to reflect fluctuating use over time and based on life circumstances and social environment, problematic use, and cessation after a period of sustained use (Hallstone, 2002; Hathaway, 2004; Hirsch et al., 1990; Järvinen & Ravn, 2014). As this literature suggests, using Becker's work to study and theorize recreational cannabis use presents some gaps and limitations given changes in social context and, in several jurisdictions, changes in legal context. However, recent studies applying Becker's work to the experience of using cannabis for medical purposes (Athey et al., 2017; Lankenau et al., 2018) offer promising findings for further research and opportunities for theorizing.

The body of research applying Becker's work to the experience of using cannabis for medical purposes is limited but it is conceptually and empirically rich. Notably, two important qualitative studies conducted by researchers in Canada (Athey et al., 2017) and the United States (Lankenau et al., 2018) suggest that medical cannabis users undergo a process of becoming of their own and report career stages that reflect the uniqueness of their experience. In both studies, medical cannabis users described a process of becoming that started with them discovering that cannabis could have therapeutic (i.e., medical) effects in addition to pleasurable (i.e., recreational) effects. In other words, before moving into a comprehensive process of social learning to become medical cannabis users, which we detail next, they had first experienced some form of therapeutic relief while using recreationally. This was reiterated in a recent study conducted in Denmark (Kvamme, 2022). From there, documented career stages from *discovering* to *becoming* vary but the common denominator is the importance of social learning in the context of medical cannabis. As noted by Athey and colleagues (2017), “the fact that users are pursuing symptom relief rather than (or in addition to) intoxication is another complexity that necessitates a more intricate learning process” (p. 226). Based on the available research, we know that while some of this learning can be done individually, through online research and self-experimenting, the majority of the learning required to become a medical cannabis user—that is to medicate effectively using cannabis and achieve optimal therapeutic relief—takes place in social contexts and through social relations. In Canada, low-threshold access points such as cannabis clubs (i.e., cannabis compassion clubs and cannabis buyers clubs) and medical dispensaries have traditionally provided the environment for this learning to take place and for these relations to form (e.g., relations with staff and peers). This has been clearly documented in the literature on medical cannabis use (see Gagnon et al., 2023), beyond the literature pertaining specifically to Becker's work which we reference above. Having access to this environment is important because, as noted above, learning how to medicate with cannabis is far more complex than learning how to “get high”; it includes learning 1) which cannabis strains to use based on their therapeutic effects, 2) which cannabis products to purchase based on symptoms, relief sought, time of day, and dosage, 3) how to consume these products to maximize therapeutic effects and minimize harms, 4) what works for others with the same health conditions and/or symptoms, and 5) how to navigate cannabis laws, policies, and programs (e.g., medical program) (Athey et al., 2017; Lankenau et al., 2018).

It is important to note, however, that social learning is not enough to *become* a medical cannabis user. As Lankenau and colleagues (2018) point out in their study, accessing cannabis in a sustained manner played an important role in participants identifying themselves as medical cannabis users and using cannabis in a therapeutic manner. For without sustained access, therapeutic relief will be occasional and unlikely to meet the needs of someone who wants to actively medicate with cannabis. Yet, access has not been a main focus of research drawing on Becker's work. Our paper seeks to address this gap by exploring the role of low-threshold access points in providing opportunities for social learning *and* sustained access to medical cannabis. Borrowing and adapting from Mofizul Islam and colleagues (2013), we define low-threshold access points as those that aim to reduce barriers (“thresholds”) to access medical cannabis, providing storefront services and products, offering onsite staff education and support, facilitating socializing and learning, centering the experience of medical cannabis users, and implementing compassionate policies and practices. In presenting the findings of a qualitative case study low-threshold access points in British Columbia (Canada), we seek to further theorize the process of becoming a medical cannabis user and situate that experience in the broader socio-political-legal context of medical cannabis in Canada. We start the paper by providing an overview of the case and detailing our methodological approach. We then move to present our findings and discuss their implications.

## The Case: Low-Threshold Access Points in British Columbia

Prior to cannabis legalization, which came into effect in October 2018, British Columbia represented an interesting case study for documenting and analyzing various aspects of cannabis production, distribution, regulation, consumption, and culture (for example, see Boyd & Carter, 2014; Mulgrew, 2006). Described as the “cannabis capital” of Canada, the province served as a real-world laboratory to understand what happens when attitudes toward cannabis change, when the supply of and access to cannabis grow over time, and when demands for enforcement and regulation increase across various jurisdictions (i.e., municipal, provincial, and federal governments). In other words, the province offered a rich milieu to understand what became the precursors of cannabis legalization, and eventually, what cannabis legalization was designed to achieve (i.e., respond to normalization, regulate supply and access, and eliminate all unsanctioned cannabis markets and access points). As the birthplace of grassroots medical cannabis in Canada (Capler & Bear, 2023; Valleriani, 2022), British Columbia was also uniquely positioned because of its growing network of storefront low-threshold access points operating outside existing drug laws and policies, in a “grey area” that lasted for decades until the *Cannabis Act* came into effect in 2018. It is challenging to know exactly how many “grey area” low-threshold access points were operating in British Columbia prior to cannabis legalization, but this number is likely in the hundreds. For example, the city of Vancouver alone had 176 medical dispensaries in 2015 (Valleriani, 2018) and one compassion club serving close to 15,000 members (British Columbia Compassion Club Society, 2022). That year, Vancouver became the first city in Canada to regulate low-threshold access points in response to the growing number of medical dispensaries despite them operating outside the existing drug laws and policies (Capler & Bear, 2023).

The history of low-threshold access points in British Columbia can be divided into four periods. The first period unfolded in the 1990s as more people turned to cannabis for therapeutic relief and the need for compassionate access grew amid the AIDS crisis (Capler & Bear, 2023; Hathaway & Rossiter, 2007; Valleriani, 2022). Drawing from the compassion club model developed by Dennis Peron in California (Malott, 2009), low-threshold access points such as the British Columbia Compassion Club Society (BCCCS), the Victoria Cannabis Buyers Club (VCBC), and the Vancouver Island Compassion Society (VICS) opened their doors. The second period, in the early 2000s, saw a rise in dispensaries and legal challenges resulting in the creation of a national medical cannabis program (Capler & Bear, 2023; Valleriani, 2022). Dispensaries were serving medical cannabis users who struggled to gain access to cannabis via the national medical cannabis program, but they generally opted for a broader retail approach as opposed to a community health approach (Valleriani, 2022). Some dispensaries (i.e., medical dispensaries) adopted a hybrid retail and community health approach (e.g., confirming medical condition or authorization, offering compassionate pricing, etc.) but they were less formalized than cannabis clubs and mostly operating on the basis that cannabis is a medicine more broadly (Valleriani, 2022). The third period, starting as early as 2010 and intensifying between 2013 and 2018 (the year of cannabis legalization), was marked by a steady rise in the number of dispensaries and medical dispensaries—which contributed to the creation of Canadian Association of Medical Cannabis Dispensaries (CAMCD) and resulted in some municipalities in British Columbia introducing their own regulatory by-laws (Capler & Bear, 2023, Valleriani, 2018)—and a steady stream of studies documenting barriers to access faced by medical cannabis users (for example, see Belle-Isle et al., 2014; Capler et al., 2017; Lucas, 2012; Walsh et al., 2013) as well as several legal challenges pointing to the failures of the national medical cannabis program (for a summary, see Ng et al., 2022). With the fourth period came cannabis legalization, a new legal scheme that ended the long-standing “grey area” in which low-threshold access points had been operating for decades (Capler & Bear, 2023). Our study was conducted at this critical juncture.

In response to cannabis legalization, low-threshold access points were presented with limited options: (1) close indefinitely, (2) close and reopen under the new licensing regime, with an exclusive focus on recreational (non-medical) cannabis (i.e., enter the regulated recreational market), or (3) stay open and face enforcement by the Community Safety Unit (CSU). The CSU did not exist prior to cannabis legalization. It was created to ensure compliance and enforcement under British Columbia's *Cannabis Control and Licensing Act* (CCLA)—the legal and regulatory framework developed by the province to oversee the cannabis market (CSU, 2024). Located in the Policing and Security Branch of the Ministry of Public Safety and Solicitor General, the CSU's mission is to “prioritize public health and safety, protect children and youth, and keep the criminal element out of the cannabis industry” (CSU, 2024). In addition to responding to complaints from the public, it collaborates with government agencies provincially and nationally, law enforcement, and legal market operators to conduct its enforcement activities in person and online (CSU, 2024). The CSU can make “seizures of cannabis and equipment used in connection with a contravention of the CCLA or the regulations” (CSU, 2024). It can also charge a person with a provincial offence resulting in administrative monetary penalties and/or imprisonment (up to 12 months) (CSU, 2024). Finally, it can collaborate with law enforcement and give rise to charges associated with a criminal offence (CSU, 2024). In the months following cannabis legalization, the CSU also conducted hundreds of “education visits.” For example, of the 362 “education visits” recorded to date, 63% (226) took place in 2019 (CSU, 2024). In comparison, since 2020, the CSU has conducted on average 27 “education visits” per year (CSU, 2024). When asked to clarify what “education visits” entailed, a CSU representative explained that the goals of these visits are “to provide education and raise awareness about cannabis laws and the penalties and consequences for violating federal and provincial regulatory regimes upon initial visits to illegal operations” (A. Wali, personal communication, February 23, 2023).

The Vancouver Island Compassion Society (VICS) closed in 2019 and the British Columbia Compassion Club Society (BCCCS) closed in 2022. The VCBC remained open but it was evicted, raided three times by the CSU and it is currently in the process of challenging administrative penalties totaling $6.5 million for selling cannabis illegally (for a full description of VCBC and its experience post-legalization, see Gagnon et al., 2023). Some medical dispensaries that closed prior to legalization were able to open under the new licensing regime, which means that they have to operate like a retail store, sell cannabis products intended for recreational use, and are prohibited from providing advice to clients who may want to medicate with cannabis or implementing compassionate practices such as lower pricing for medical cannabis users. Other medical dispensaries closed but were never able to open because the new licensing regime is expensive and labor-intensive. The ones that remained open, such as the Blue Door and The Medicinal Cannabis Dispensary (TMCD) in Vancouver, were raided by the CSU. To our knowledge, only the TMCD remains open. It is worth noting that, according to the CSU website, a total of 234 “stores” were closed following “an education visit” between 2019 and 2024 (CSU, 2024). It is unclear how many of the stores visited and subsequently closed after an education visit functioned as low-threshold access points for medical cannabis users, but it is helpful to keep this number in mind to appreciate the shift in enforcement following cannabis legalization and how that changed the provincial landscape.

Prior to cannabis legalization, a majority of medical cannabis users in British Columbia accessed their cannabis at “grey area” low-threshold access points. In a study conducted by Belle-Isle and colleagues (2014), for example, 70% of participants residing in British Columbia reported accessing cannabis via low-threshold access points compared to 2% who reported accessing via the national medical cannabis program. This is consistent with other studies, which found cannabis clubs and medical dispensaries to be significantly more accessible and scoring better than the national medical cannabis program on 1) affordability, 2) product quality, safety, diversity, availability, and consistency, and 3) quality of the service (Belle-Isle et al., 2014; Capler et al. 2017; Valleriani, 2022; Walsh et al., 2013) Two main reasons explain this. First, the high number of low-threshold access points in the province. Second, the difference between a *high-threshold model* that requires medical authorization, imposes limits on all aspects of medical cannabis use, creates access barriers, and prohibits storefront access, and a *low-threshold model* that provides storefront access to education, support, community, and a range of properly dosed and compassionately priced cannabis products that can be consumed in ways that generate optimal therapeutic relief. What happens when this low-threshold model disappears? What does it means for medical cannabis users? These have been central questions in our work. In this paper, more specifically, we seek to explore the role of “grey area” low-threshold access points in British Columbia in the process of becoming a medical cannabis user, as spaces that contribute to social learning *and* sustained access to medical cannabis.

## Study Design

Qualitative case study methodology, as defined by Stake (1995, 2005, 2006), offers a flexible approach to explore and describe a particular case in a real-life setting. As Stake (2006) explains, “qualitative case study was developed to study the experience of real cases operating in real situations” (p. 3). The case can be defined as an individual, a group, a program, a city, a country, or a particular phenomenon of interest (Stake, 1995, 2005, 2006). Stake (1995, 2005, 2006) identifies three types of case studies: intrinsic, instrumental, and collective (or multiple). An intrinsic case study is undertaken to analyze a unique case and develop a better understanding of this case alone (Stake, 1995, 2005). In contrast, an instrumental case study is primarily undertaken to examine a case that can provide insights into a broader phenomenon (Stake, 1995, 2005). When this approach is extended to multiple cases, it becomes a collective case study (or multiple case study) (Stake, 1995, 2005, 2006). Case study research starts from the question: “what can be learned from the single case?” (Stake, 2005, p. 443). As such, the goal of the researcher is to understand the case as an integrated and bounded system that is located in a particular situation and in a broader context (Stake, 1995, 2006). Exploring the inside of the case while also paying close attention to what is going on outside is important when conducting this type of research (Stake, 1995, 2006). Drawing from multiple data sources data such as interviews, questionnaires, observations, documents, and field notes is, therefore, expected (Stake, 1995, 2005, 2006). Moreover, remaining responsive to the case in real-time is important and it may require the addition of new data sources or lead to the emergence of new questions. Finally, in analyzing the case, the process of identifying particularities through interpretive inquiry (as opposed to generating findings for generalizability) is what makes case study research valuable (Stake, 1995, 2006). As Stake (1995) notes, “the real business of case study is *particularization*, [through understanding of the case itself], not *generalization*” (p. 8, emphasis added).

We undertook a qualitative case study of low-threshold access points in British Columbia at the critical juncture described above. Case study methodology allowed us to situate low-threshold access points in the broader socio-political-legal context of medical cannabis in Canada while also attending to the particularities of the province; tracing the history of low-threshold access points, analyzing the experiences of people involved in operating these access points and those accessing them (i.e., members or clients), exploring the interactions between low-threshold access points and structural factors such as policies, laws, and programs, and piecing together a picture of the province before and after cannabis legalization. For reasons we detail above, it was more appropriate to study low-threshold access points in British Columbia as an instrumental case study rather than an intrinsic case study. Instrumental case study allowed us to dive into the broader phenomenon of low-threshold access in the context of medical cannabis and how this type of access has been achieved historically—and how cannabis legalization has radically changed this. Using the province with the highest concentration of low-threshold access points in Canada was a good starting point. To study the case and situate it within the broader socio-political-legal context of medical cannabis in Canada, we spent six months collecting data across multiple sources and another six months engaging with the data.

### Data Collection

**Figure 1.**
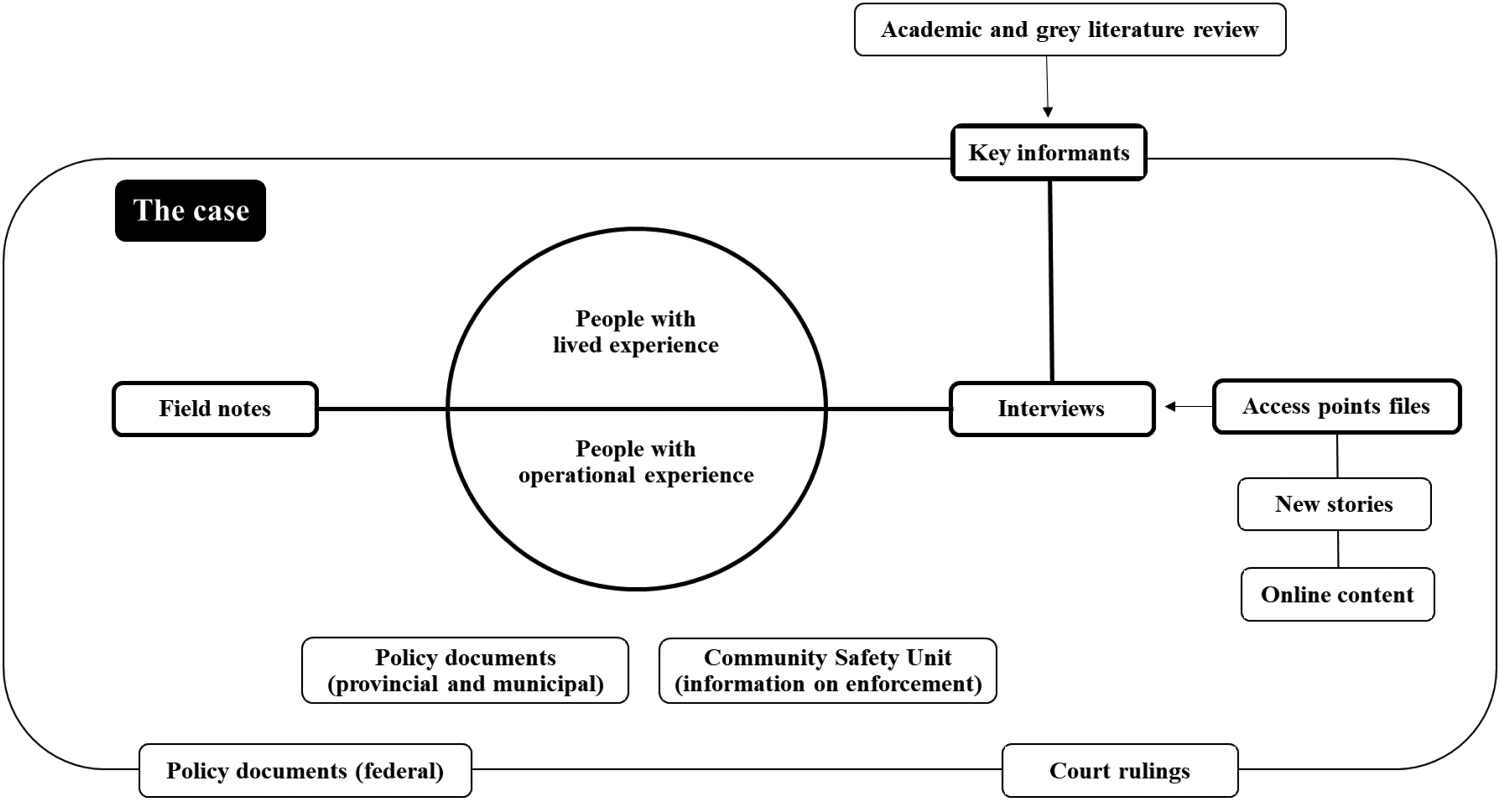
Overview of the case study sources.

In total, we included seven data sources (see Figure 1): (1) online content, (2) news stories, (3) legal documents, (4) policy documents at the federal, provincial, and municipal levels, (5) information about the CSU and its enforcement activities, (6) interviews with (i) key informants, (ii) participants with operational experience (i.e., people engaged in the active operations of low-threshold access points in different capacity), and (iii) participants with lived experience of medicating with cannabis, and finally (7) field notes. After obtaining harmonized ethics approval from the University of Victoria and the University of British Columbia, we completed a series of advanced Google searches to locate news stories mentioning low-threshold access points. We found a total of 86 news stories that mentioned 37 low-threshold access points in British Columbia. We also searched *Cannabis Digest* and *Cannabis Culture Magazine*, two popular websites that publish entries related to cannabis, which respectively generated 209 entries referencing 29 low-threshold access points and 114 entries referencing 10 low-threshold access points. Using the above web searches and removing duplicates, we cataloged 55 unique access points and extracted information to create a file for each access point (e.g., history, names and contact of people involved, services provided, etc.). In addition to this, we conducted a search to identify all relevant policy documents, legal documents, and enforcement activities of the CSU.

Drawing on the literature review we completed in preparation for the study and the above searches, we created a list of potential key informants with known expertise in one or several domains (e.g., law, research/scholarship, advocacy, policy-making, enforcement) and sent an email invitation asking them to participate in a one-on-one virtual interview to contribute contextual information about the case. We interviewed a total of 11 key informants who agreed to participate and consented orally to taking part in the study, tailoring the interview themes (see Table 1) to each key informant based on their domain(s) of expertise. These interviews were summarized using time stamps and short descriptions of the main ideas discussed. They were not transcribed or coded because key informants were asked to shed light on the broader socio-political-legal context of medical cannabis in Canada as a means of situating the case. Then, using our access point files, we emailed potential participants with operational experience and recruited additional participants through snowball sampling. We interviewed a total of 15 participants with operational experience (five in person and 10 by phone) with the goal of understanding their experience with low-threshold access points, contextualizing that experience before and after legalization, and exploring access to cannabis as a medicine (see Table 1). All of the participants consented orally to taking part in the study after reviewing the consent form. After completing this round of interviews, we recruited people with lived experience via the VCBC and TMCD and completed 12 phone interviews, each preceded by the completion of a short socio-demographic and cannabis consumption questionnaire. This round of interviews focused primarily on the experiences of medicating with cannabis and accessing low-threshold access points before and after legalization (see Table 1). All of the participants consented orally to taking part in the study after reviewing the consent form. Finally, we recorded field notes throughout the data collection process, including during visits at VCBC and at gatherings where members provided testimonials on the importance of low-threshold access points to media and policy-makers.

**Table 1.** Outline of Inclusion Criteria and Interview Questions.

Group and inclusion criteria	Interview themes (key informants)
	Interview questions (participants)
*Key informants*	
Inclusion criteria Identified^a^ as someone with expertise in one or several of these domains: Law and/or Research/Scholarship and/or Advocacy and/or Policy-making and/or Enforcement	*We tailored the questions to the domain(s) of expertise of each key informant.* Core themes and sub-themes: Theme 1: Access points in BC, *pre-legalization* History and specificities of BC and its LTAPs Context in which these LTAPs operated pre-legalization Role of LTAPs for people who use CTP Theme 2: Access to cannabis as a medicine, *pre-legalization* History and specificities of use of/access to CTP in BC Challenges faced by people who use CTP Profile of people who use CTP in BC Theme 3: Access points in BC, *post-legalization* Uniqueness of BC in comparison to other jurisdictions Impact of legalization on LTAPs in BC Consequences of closing LTAPs Challenges faced by LTAPs that remain open Theme 4: Access to cannabis as a medicine, *post-legalization* Impact of legalization on people who use CTP Thoughts on remaining access to cannabis for people who use(d) LTAPs and have therapeutic needs Consequences specific to people who use CTP Concluding questions What works/does not work in BC's approach to LTAPs? What is missing or could be done differently? What gaps exist for people who use CTP? What documents should we add to the case study?
*People with operational experience*	
Inclusion criteria 19 years or older Residing in BC Able/willing to complete interview Direct experience of opening and/or running a LTAP (or LTAPs) • LTAP(s) meet(s) definition^b^ • LTAP(s) located in BC • Direct experience in role(s): • Founder (or co-founder) • Management or leadership • Governance (board of directors) Frontline staff or volunteer Grower/baker/producer	*After answering three pre-interview questionnaire (i.e., type of LTAP(s), roles, and years of experience), participants were asked the following questions. We tailored the questions to the stated LTAP(s) and role(s):* Introduction and background Please tell me about your work as it relates to cannabis, including anything you think is important for me to know about and would be helpful to set the stage for our discussion. Access point, role, and raison d’être Can you describe how, why, and when you got involved with [type of LTAP]? Can you explain in a bit more details your role and what your involvement looks(ed) like? In your own words, what is(was) the goal of this LTAP? Who does(did) it serve? In your experience, why was this type of LTAP needed? Situating the experience on context, *pre-legalization* When you became involved with this LTAP, where there other similar access points in BC? What was going on in the province? Can you describe the general context in which your LTAP operated before cannabis legalization? Can you describe the local and provincial context? What challenges did you face? What opportunities did you encounter? What was your experience with policing and the criminal justice system? Going into legalization, how was the LTAP affected? Were any measures taken to keep the LTAP open, close it, protect it somehow? Going into cannabis legalization, did you personally have any hopes and/or worries? Situating the experience on context, *after legalization* Can you describe what happened to your LTAP after cannabis legalization? *If it remained open*: How would you describe the local and provincial context post-legalization? What challenges did you face? What opportunities did you encounter? What was your experience with policing and the criminal justice system? What was your experience with the cannabis enforcement system? *If your site has closed*: Can you walk me through the steps leading to the closure? Did legalization contribute to the closure? How? Can you describe who was left behind following the closure? Access to cannabis as a medicine, *pre- and post-legalization* Based on your experience, what was the experience of people who use CTP before legalization? Access Products Challenges Opportunities What is the experience of the same people now, post-legalization? Has anything changed? Can you explain why LTAPs have (historically and in BC) been important to people who use CTP? Can you describe the differences between the current government-sanctioned stores and LTAP from the perspective of therapeutic users? Concluding questions What works/does not work in BC's approach to LTAPs? What is missing or could be done differently? What gaps exist for people who use CTP? What documents should we add to the case study?
*People with lived experience*	
Inclusion criteria 19 years or older Residing in BC Able/willing to complete interview Lived/living experience of chronic illness and/or chronic symptoms Lived experience of accessing a LTAP (or LTAPs): • LTAP(s) meets definition^b^ • LTAP(s) located in BC • Use of CTP as defined by participant	*After completing the pre-interview questionnaire (*see Tables 2 and 3*), participants were asked the following questions:* Introduction and background Please tell me about your experience with cannabis, including anything you think is important for me to know about and would be helpful to set the stage for our discussion. Cannabis use and access at LTAP (as defined below) Can you describe why and when you started using CTP? Can you explain how cannabis is therapeutic for you? How do you use it? What works for you? I understand that you have experience accessing cannabis at a [type of access point]. Can you tell me more about that experience? Why are(were) places like [type of access point] important to you as a person who uses CTP? What are the challenges faced by people who use CTP in accessing cannabis as a medicine? Access points before and after legalization Can you describe your experience accessing a [type of access point] before cannabis legalization? Has anything change since legalization? How so? How has legalization affected you as someone who uses cannabis as a medicine? Concluding questions What works/does not work in BC's approach to LTAP? What gaps exist for people who use CTP?

*Note.* LTAP(s)= low-threshold access point(s); CTP = Cannabis for therapeutic purposes; BC = British Columbia.

^a^ Potential key informants were identified during the literature review, online content search, media search, and case law review (see Figure 1).

^b^ In this study, we define *low-threshold access points* (LTAP as abbreviated above) as sites/organizations/groups that provide (or provided, if now closed) access to cannabis to people who live with chronic illnesses and/or chronic symptoms for symptom relief, wellbeing, substitution of prescribed medications, self-medicating, traditional healing, etc. Examples include cannabis compassion clubs, cannabis buyers’ clubs, and cannabis medical dispensaries.

### Data Analysis

**Figure 2.**
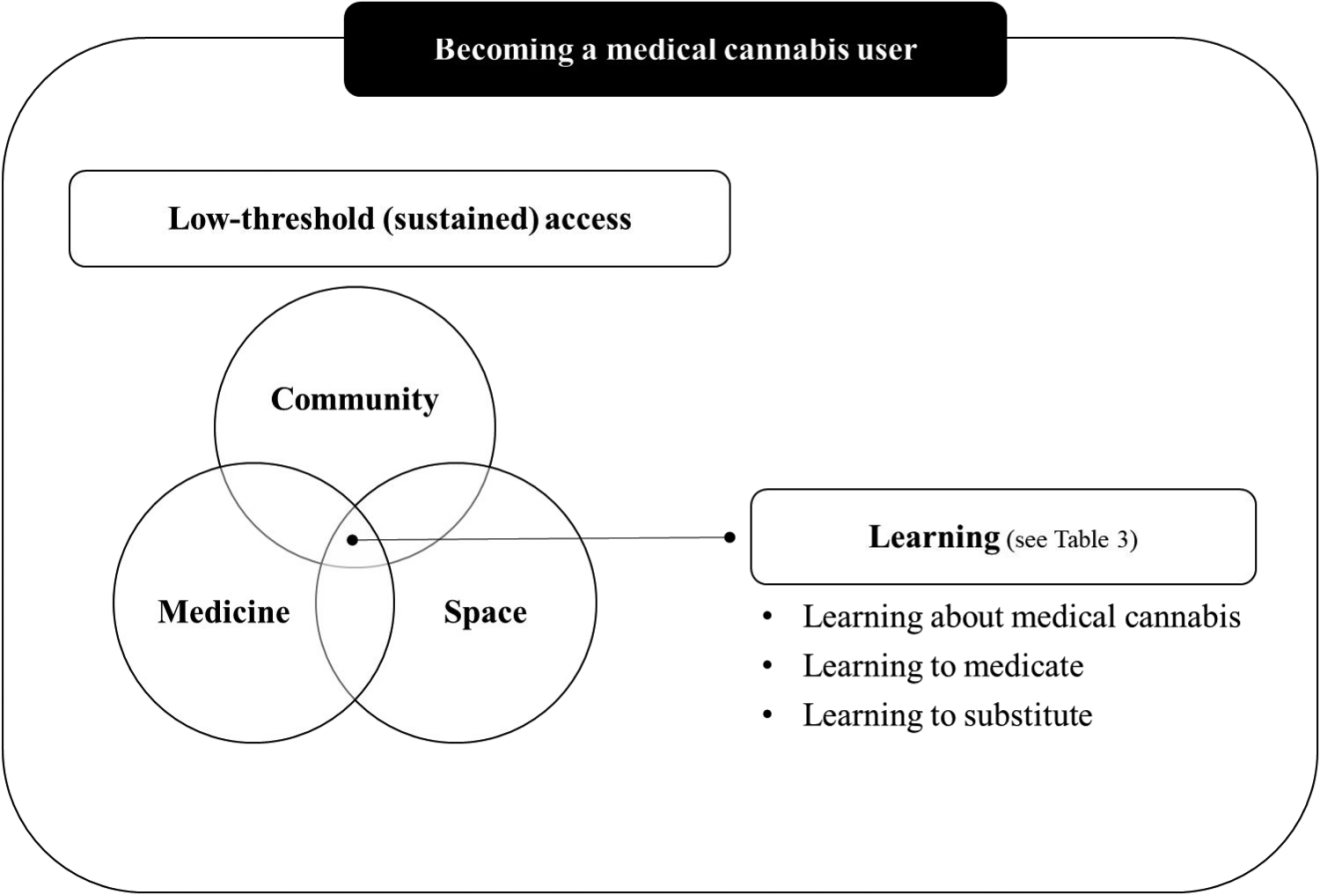
Summary of findings.

To organize our case study, we started from the inside of the case with the transcribed interviews of participants with operational and lived experiences and moved toward the outside of the case where key informant interviews and search results provided context to situate low-threshold access points over time and policy eras (see Figure 1). We also worked with the entirety of the data, identifying a need to analyze the interview sets separately as well as the need to conduct the theory-informed analysis we present in this paper. After reviewing and summarizing the extent of the literature on Becker's work and subsequent application to medical cannabis, we developed a three-part matrix and organized the extracted data under the broad themes of (1) social learning, (2) career stages, and (3) formal and informal social controls. From there, we used Applied Thematic Analysis (ATA) (Guest et al., 2011) to (1) code the “social learning” data, (2) identify possible themes, (3) compare and contrast themes, identifying structure among them, and (4) produce a thematic scheme that can generate new theoretical insights (see Figure 2). The content included under career stages and social controls were not coded because of the stated scope of this theory-informed analysis.

## Results

The majority of participants with lived experience were 50 years or older, had an annual income of less than $30,000, listed disability assistance as their primary source of income, and were renting a room or an apartment (see Table 2). It is important to note, however, that the age of our participants did not reflect the age at which they had started medicating with cannabis nor the age at which they started accessing low-threshold access points (the majority operational for 10–20 plus years prior to legalization). For many participants, the symptoms and health conditions for which they were medicating had started earlier in life. We did not systematically collect this information in the pre-interview questionnaire. However, as participants shared their experiences, they did provide an overview of their journey with medical cannabis spanning over decades in most cases. Educational level was distributed across the sample and gender representation was close to balanced with seven cisgender women and five cisgender men. All of the participants identified as White (European descent). In terms of cannabis consumption (see Table 3), the majority of participants reported using daily (including multi-daily) and all of the participants indicated using cannabis for therapeutic purposes and for their own (physicial/mental) wellness. Some participants listed additional reasons, the most common one being recreational and social use (*n* = 4). Their preferred modes of consumption, included ingesting edible products (*n* = 10, 83%), smoking a joint without tobacco (*n* = 5, 42%), and applying on the skin (*n* = 3, 25%). Working through a theory-informed analysis of this set of interviews, we were able to identify a core structure that speaks to Becker's work and reflects the main themes across the data (see Figure 2). Together, these themes suggest that becoming a medical cannabis user is a process that requires low-threshold (sustained) access to community, medicine, and space—three domains across which learning unfolds, in relationships (with staff and peers) and in response to health and social needs.

**Table 2.** Characteristics of Participants with Lived Experience (*n* = 12).

	*n* (%)
Age (years)	
31–40	3 (25)
51–60	5 (42)
61–70	2 (17)
>71	2 (17)
Gender	
Cisgender man	5 (42)
Cisgender woman	7 (58)
Ethnicity	
European descent (White)	12 (100)
Housing	
Unhoused	1 (8)
Renting (room or apartment)	8 (67)
Owning (condo or house)	3 (25)
Income	
$10,000-$19,999	6 (50)
$20,000-$29,999	4 (33)
$30,000-$39,999	1 (8)
Unknown	1 (8)
Sources of income^a^	
Income assistance	8 (66)
Full-time employment	1 (8)
Part-time employment	2 (17)
Pension/employment insurance	2 (17)
Highest level of education completed	
Less than high school	4 (33)
High school	1 (8)
Registered trade or apprenticeship certificate or diploma	1 (8)
College	2 (17)
University (undergraduate – bachelor's degree)	2 (17)
University (undergraduate – master's degree)	2 (17)

^a^ Select all that apply. Participants could report multiple sources of income.

**Table 3.** Cannabis Consumption Reported by Participants with Lived Experience (*n* = 12).

	*n* (%)
Frequency	
Multiple times a week	1 (8)
Once a day	2 (17)
Multiple times a day	9 (75)
Reasons^a^	
For therapeutic use for a chronic illness or chronic symptoms	12 (100)
For your own physical and mental wellness	12 (100)
For recreational use (fun) or social use	4 (33)
For spiritual use or traditional medicine purposes	1 (8)
Other: *for additional benefit of being more creative*	1 (8)
Preferred mode of consumption^a^	
Ingesting edible products	10 (83)
Smoking joints without tobacco	5 (42)
Smoking using a pipe	2 (17)
Vaporizing and vaping	2 (17)
Applying on the skin	3 (25)
Using suppositories	1 (8)

^a^ Select all that apply. Participants could report multiple reasons and modes of consumption.

### Accessing Community, Medicine, and Space

#### Accessing Community

Participants explained that accessing a community centered around the creation and sharing of knowledge played an important role in the process of becoming a medical cannabis user. Community was made up of staff working in low-threshold access points, many of whom had lived experience of medicating with cannabis as well as professional experiences working in the field of cannabis in various roles (e.g., intake, sales, production, management), and peers who had acquired knowledge through their own experience and their interactions with others.

Yeah, ‘cause I missed a major point, and that is the community. The community. So many times, I’ve said to [name], there is not a better university I could have gone to on the planet. Ok, I’m not saying I know everything about everything; I don’t. I’ve learned so much though, because of the people that I have met since the nineties (…). And not only have I learned about, you know, cannabis as a medicine and the politics surrounding it and why it got turned illegal in the first place and so many things! But I’ve met so many beautiful humans and each one of us is a gold mine of knowledge. (Participant 10, lines 210–218)

By acting as a knowledge community, low-threshold access points allowed participants to learn in ways that were not available through the health care system nor the medical cannabis program or the licensed retail stores opened post-legalization, as we note below. When asked why accessing knowledge in community was important to them as medical cannabis users, participants explained that there is a clear connection between learning *and* medicating.

(…) The knowledge of these people, [name of access point]'s crew, was indispensable! They knew stuff that I never dreamed of. They introduced me to the Cannoil^1^ products and they had more compassion, knowledge and guidance than my pharmacy manager or doctor, literally. Quite literally, I put them above the medical institution and the pharmacies. Much, much higher in value.

*Interviewer: In terms of the kind of information or the depth of information?*

Both. Both. Yes. I didn’t know what CBD was. They guided me through that. And I didn’t know what Cannoil was so, the depth of information, the variety of information, the um, advice and dosage, all of it. I couldn’t believe that [name of access point]'s crew was privy to this knowledge. It blew my mind. (Participant 2, lines 126–135)

In addition to providing access to knowledge, low-threshold access points also generated knowledge by showing an interest in the illness and symptom experiences of their clients or members, actively collecting feedback on the effectiveness of available products, and summarizing this experiential knowledge to provide guidance and support for other clients.

(…) they’re keenly interested in what you’re specifically, you know your pain problems are and what your issues are and they, you know they’re interested in your feedback, like what has worked for you so that they can share information with other people. They’re careful not to give medical advice obviously, but they do want to hear back about whether what they’re doing is working, is helping people. (Participant 1, lines 179–184)

Being able to access this community-driven knowledge was an important part of becoming a medical cannabis user because all of our participants agreed that medicating with a plant involves some level “experimentation” (i.e., trying, reporting, adjusting, etc.) and “discovery” (i.e., achieving therapeutic relief with the right products, dosage, scheduling, etc.). Participants placed a lot of value on community-driven knowledge because it was generated from the experiences of peers (i.e., people with similar illnesses and symptoms) and addressed existing gaps in knowledge across the health care system. Participants were unanimous that, based on their experiences, health care providers were not knowledgeable about cannabis and had not played a role in their process of becoming (educated) medical cannabis users. They credited low-threshold access points for filling this knowledge gap.

But anybody, like, physicians, nurses or others I’ve discussed with are generally supportive of it, but they really don’t know much about it. And uh, like I know more about it, educating myself from hanging out at [name of access point], than medical professionals do, really. That's what I perceive. Any medical professional I’ve talked about it to, I try to approach a few about it and, they’re generally supportive but they just don’t know anything about it (laughs). About cannabis and its benefits. (Participant 5, lines 252–261)

They also explained that legalization had resulted in the dismantlement of low-threshold access points (with the exception of VCBC and TMCD) and, as such, decreased access to knowledge. A number of participants explained that the only storefront access left in the community were licensed retail cannabis stores, which are not only prohibited from providing education and support to medical cannabis users but are also staffed with people who are new to cannabis and hired into retail jobs.

(…) generally I’d say no [medical cannabis users can’t access the information they need] because when I go to most of these retail stores, it just seems like it's like, you know, some young twenty year old working a minimum wage job (laughs). You know, like if I’m like, hey um, “I want something to help me sleep” they’re just like, “oh here's an Indica”, you know? (laughs). I’m like “well no-no-no, like I KNOW that, but like can you be more specific?” “No I can’t, I started this job two months ago, you know?” (laughs). (Participant 4, lines 276–281)

The above quote helps to illustrate that becoming medical cannabis users is a communal rather than an individual process. In other words, learning from knowledgeable people (staff and peers) and being able to access, co-create, and share knowledge in community had played a crucial role in transforming cannabis into a medicine that participants could use to achieve effective symptom relief. Accessing the “right medicine,” which we explore next, was also crucial but as noted in one of the above quotes (see Participant 2), accessing community had paved the way for them to be able to access the right medicine and become medical cannabis users.

#### Accessing Medicine

Throughout the interviews, it was evident that achieving therapeutic relief with cannabis in a way that is effective, reliable, and consistent was a defining feature of becoming a medical cannabis user. However, this was only made possible by accessing the “right medicine” (i.e., the right product(s), at the right dosage, consumed at the right time, using the right mode of consumption) in a sustained manner, which was an important reason why participants relied on low-threshold access points as opposed to buying off the underground illegal market or at licensed retail stores after cannabis was legalized. Several participants explained the difference between accessing any kind of cannabis and accessing cannabis as a *medicine* (i.e., a substance that can provide therapeutic relief). They agreed that, in order to medicate with cannabis, you have to know what cannabis to look for, what you are using, and how. As one participant describes:

[buying off the street] was hit-and-miss, you didn’t always know that people were, would be there. And, lots of times it was really good pot and just, TOO strong sometimes. You know and, at [name of access point] you get to choose and ask questions about the pot, you know? And the other, buying off the street you just, get what you’re given. It was a reasonable deal, most of the time I got really good pot for ten bucks a gram so, no complaints (laughs). But it was just, you got the, you didn’t know what you were, what strain you were getting, whether it would be an Indica or a Sativa or how strong it would be. You know, you didn’t really know what to expect until you consumed it. (Participant 5, lines 169–177).

Being able to access cannabis in a reliable and consistent manner was also part of becoming a medical cannabis user. However, as noted by participants, the introduction of a mail-order medical cannabis program in the early 2000s and the opening of licensed retail stores following legalization in 2018 did not improve their access. Major barriers persisted because medical cannabis users need *storefront access* to a *consistent supply* of *high quality cannabis products* selected, developed, and packaged for *medical use*, and *priced compassionately* for medical cannabis users on a limited/fixed income who do not have any medical coverage. Before they were closed, with the exception of VCBC and TMCD as indicated above, low-threshold access points in British Columbia helped overcome these barriers.

(…) there was a couple of other [access points] that are shut down now that sort of made the point of providing good quality edibles and other products like salves, suppositories, things like that but at affordable prices so that people were, you know, so like a lot of high-dose stuff, like unless people are cutting it up in smaller bits, it's not stuff that people are going to use recreationally. (Participant 3, lines 101–107)

According to all participants, what characterized low-threshold access points was the quality, diversity, and consistency of their cannabis products, the available dosages, and the desire to innovate by developing new products and provide access to existing products that are intended for medical use (i.e., products that have “no recreational value,” as noted by several participants including Participant 3). Low-threshold access points had made it possible for participants to access not any kind of cannabis, but the kind that they could medicate with. One participant who medicated with Rick Simpson oil (RSO) three times a day for chronic pain summarized it as such:

I am a firm believer, after many years of using [cannabis] myself and also listening to others, that the most effective way to get pain relief, physical pain relief is to use edible forms of cannabis and topical forms of cannabis. And so I do, three times a day. I use something that is called, the nickname is RSO, stands for Rick Simpson, is the man who invented a highly concentrated, very clean form of cannabis, edible cannabis. And (…) I purchase it at [name of access point] (…). It is such a game-saver (…) And this is all coming out of my pocket, on my very limited income so it's very important to me to get, to be the *most effective with my dosing*. So RSO is the way to go for that. (Participant 10, lines 58–77, emphasis added)

The idea of “*being effective with dosing,*” as noted in the above quote, reiterates why participants accessed their medicine at low-threshold access points. In addition to having access to a community and a supply of high-quality cannabis products intended (and priced) for medical use, they were able to find the medicine that worked best for them and generated the most relief in the most inexpensive form possible. As such, cost-effectiveness was a major consideration when accessing medicine and an important reason why participants credited low-threshold access points for being able to medicate with cannabis. It was clear that becoming a medical cannabis user was conditional upon being able to afford the medicine in the first place. Participants were unanimous that affordability from the perspective of medical cannabis users is part of access and that having products available in licensed retail stores post-legalization, for example, did not provide them with more access nor the ability to “*being effective with dosing.*” As one participant explained:

I would say, it was nice how before cannabis got legalized and enforcement started taking place around some of these grey market access points, you could actually access edibles with a dosage high enough to be of use to someone like me, medically. I can’t afford to, nor do I want to buy, five packages of edibles every time I want to eat a fifty milligram dose because they’re not allowed to sell more than ten milligrams in a package legally. (Participant 11, lines 185–193)

In addition to the importance of accessing community and medicine, participants highlighted the importance of accessing a physical space in the process of becoming a medical cannabis user. As we detail next, having access to a storefront space and a consumption space helped with learning and medicating in addition to creating opportunities for socializing.

#### Accessing Space

Accessing space, and more specifically a storefront space, was described as a core component of becoming a medical cannabis user since it requires learning from and talking to staff and peers, exploring various medical cannabis products (e.g., looking, touching, smelling, tasting, sampling), having a place to go for support and advice, and being able to visit based on ability to purchase or store products (e.g., number of visits and amounts purchased based on limited income and housing status). As such, storefront access played an important role in lowering the threshold to medical cannabis, as one participant explained:

(…) I walk through that door and I’m served immediately. Not forms and mail orders and waiting for it to arrive, you know, it's there, immediately when I need it. Not some federal program that requires fields of interest being properly filled out and the bureaucratic nightmare of waiting and jumping through their hoops and on their agenda, on their time schedule. Daylight and darkness. (Participant 2, lines 336–340)

Storefront access is what made low-thresholds access points so important to medical cannabis users. These access points addressed a long-standing gap for people wanting to self-medicate with cannabis and people for whom the medical cannabis program did not offer the cannabis products they needed for therapeutic relief nor the steady supply of such products. Participants who had secured a medical authorization, in particular, noted how important storefront access had been for them and why the mail-order medical cannabis program fell short of meeting their needs as medical cannabis users. Prior to cannabis legalization storefront access had not been difficult for medical cannabis users.

Well there were even so-called medical marijuana places back before legalization too. They were all over the place, they even had them downtown, they had them on the [name of street]. [name of access point] was one of the places operating under the grey zone of medical marijuana before it went legal. So before it went legal there were a lot of places where you could get it. (Participants 9, lines 99–103)

However, because cannabis legalization ended the long-standing “grey area” in which brick-and-mortar low-threshold access points operated, the only storefront spaces remaining at the time of the interviews (with the exception of VCBC and TMDC) were licensed retail stores, which are prohibited from selling cannabis for medical purposes and not intended for medical cannabis users. As one participant explained, walking into licensed retail stores “feels like I’m a product to them, or you know, like I’m just a way for the company make money and not like a person who's here for medicine.” (Participant, 4, lines 226–227).

In addition to reflecting on the importance of storefront access in the process of becoming a medical cannabis user, participants spoke of the importance of accessing a consumption space. Being able to sit with peers in a space where cannabis can be consumed safely created opportunities to learn from others and medicate more effectively.

(…) people were just sitting in there and everybody's talking, so somebody else has rheumatoid arthritis, we start talking, like what works for you and what's your, you know like, people get information from other clients too (…) sitting with somebody that has the same issue as you, it's like, oh we’re in the same boat together. (Participant 6, lines 183–199)

For participants who were unhoused and precariously housed in multi-dwelling units (e.g., rooms, apartments, condos), in particular, being able to smoke/vape/vaporize in a dedicated and protected space was paramount. Numerous participants mentioned that smoking/vaping/vaporizing had become more difficult following legalization because of the introduction of new rules, the closure of low-threshold access points with consumption spaces, and the absence of legal consumption spaces. One participant described the shift from being able to smoke/vape/vaporize at home to being confronted by their landlord. While this participant was able to walk to another location, this was not reflective of all participants, especially those with limited mobility and those accessing public spaces where smoking/vaping/vaporizing is prohibited in British Columbia.

Well up until I started to get confronted by the landlord, I used to go out onto my balcony, it's a third floor but in the last while, I walk down toward the beach (…). (Participant 8, lines 40–42)

Accessing a consumption space also helped participants identify as *and* identify with medical cannabis users, which in turn, helped break from the isolation they experienced as people living with chronic illnesses, symptoms, and disabilities. This compelling quote clearly illustrates this:

(…) I started going to [name of the consumption space] and I got to know all these people like I dunno, twenty or thirty different people, that I hadn’t known before and they were all kind of in similar circumstances as me. A lot of them had internal problems that weren’t um, you didn’t know what their problem was, you know? Or they had disabilities or mental disabilities, and I was thinking “oh wow, look at all these people, they’re trying to get by too, they’re on disability too, they’re you know, trying to buy pot and they got no money (laughs) and somebody helps them out”. And I experienced all that at [name of the consumption space], and it was wonderful because (…) when you’re on disability you kind of have to cobble a network of friends together for yourself, out of other disability people (laughs). But like that's where [name of the consumption space] was indispensable in helping me do that and helping me, you know, develop my social skills. With a whole bunch of people, like the place was really busy there, many many times and you went in there it was just a cacophony of sound. You somehow fitted yourself in and have a conversation with somebody because somebody forced you to talk to them, you know? (laughs). It really, it was a community (…) It's people's living room. (Participant 5, lines 104–123)

To conclude this section of the findings, the interviews revealed that becoming a medical cannabis user is a process that requires low-threshold (sustained) access to three interconnected domains that we identified as community, medicine, and space. Furthermore, our findings show that this process is inherently social. Low-threshold access points contributed to this process in various ways; by providing medical cannabis users with a place to go and a place to be in community, facilitating and encouraging social learning, removing access barriers to medical cannabis products that have been developed and priced for people who experience chronic health conditions, and recognizing that medicating with cannabis is a guided practice as much as it is an embodied and relational experience.

### Learning Across the Three Domains

Social learning was part of becoming a medical cannabis user. Even the most knowledgeable and experienced recreational cannabis users among our participants emphasized that learning to medicate with cannabis is a lot more complex than it may seem. They also noted that discovering therapeutic benefits while using cannabis for recreational purposes did not amount to knowing enough to medicate effectively or understanding the potential benefits of using cannabis for medical purposes.

So that's what I got out of [name of access point], I met good friends and I just got educated about the actual medicinal benefits, ‘cause I didn’t know anything before that really. I knew there was medical benefits but it was, I had only used cannabis recreationally before 1997. (Participant 5, lines 157–160)

It is important to reiterate that learning in the context of becoming a medical cannabis user was never discussed on its own. It was always situated in the context of having low-threshold (sustained) access to community, medicine, and space. For example, one participant who was an experienced recreational cannabis smoker explained that gaining (sustained) access to edibles via a low-threshold access point is what created the conditions for them to learn how to manage pain and side effects with edibles and eventually how to substitute prescription opioids. This participant knew, from experience, that edibles “felt good” but needed the right combination of low-threshold access *and* learning to become a medical cannabis user.

(…) it was in 2007 because my teeth were in really bad shape and I was on really strong, under a doctor's supervision, I was on really strong opiates and the side effects were really harsh and I was smoking cannabis to deal with it and the odd time I’d run into an edible from someone, like a cookie and I would feel good, like REALLY good for about a day or two (…) but they weren’t readily available in the black market (…) so that's why I decided to go to the [name of access point] (…) because I really did see the value of the edibles at that point so that was about 2007, when I started taking edibles on a regular basis and using them initially just for side effects and then, you know, for substituting for opiates as well. (Participant 3, lines 31–45)

To illustrate the contribution of low-threshold access points to the process of becoming a medical cannabis user, we grouped the broad areas of learning described by participants into three main categories of learning: 1) learning about medical cannabis, 2) learning how to medicate, and 3) learning how to substitute. We summarize each category in Table 4, focusing primarily on synthesizing the breath of knowledge and skills participants had gained by accessing community, medicine, and space and documenting the contribution of low-threshold access points.

**Table 4.** Categories of Learning.

Learning about medical cannabis	Learning how to medicate	Learning how to substitute
Anatomy the plant Types of plant and stains Therapeutic components Medical properties Types of products Dosages Modes of consumption Therapeutic benefits based on symptom and/or illness Potential adverse effects Quality assessment Pricing Medical access	Dosing effectively PRN^a^ regimen Daily regimen Combining products Consuming products Reducing potential harms (e.g., ingesting vs smoking) Maximizing desired effects Minimizing adverse or undesirable effects Defining therapeutic relief and therapeutic goals Monitoring symptoms/illness Rationing and cost-saving	Substituting pharmaceuticals Partially Fully Substituting one cannabis product for another Substituting one mode of consumption for another

^a^ PRN stands for *pro re neta*, which translates to “as the need arises.”

## Discussion

The main objective of this study was to explore the role of low-threshold access points in the process of becoming a medical cannabis user. As noted in the introduction, we approached this study with an understanding that one becomes a medical cannabis user by learning how to medicate effectively with cannabis and that low-threshold access points, such as cannabis clubs and medical dispensaries, have traditionally provided an environment for this learning to take place in relationship with staff and peers. We also noted gaps in Becker-related research, mainly that focusing on social learning without also looking at the social and structural conditions for this learning to take place is too narrow and overlooks important aspects of the process of becoming a medical cannabis user. By exploring how these low-threshold access points contributed to the process of becoming a medical cannabis user, we were able to generate two main findings. First, we determined that becoming a medical cannabis user is a process that is situated and experienced at the intersection of low-threshold (sustained) access to community, medicine, and space. Second, we found that social learning, across three categories, required low-threshold (sustained) access to community, medicine, and space. In other words, that this type of learning and this type of access go hand in hand in the process of becoming a medical cannabis user.

While some of our findings echo the findings of previous research conducted on low-threshold access points, they also differ because of the current socio-political-legal context of medical cannabis in Canada. Consistent with other studies conducted on cannabis clubs and medical dispensaries in North America and Europe, we found that these low-threshold access points promote social learning in addition to providing support, promoting wellbeing, and mimizing risks and harms (Athey et al., 2017; Belackova et al., 2016; Belle-Isle et al., 2014; Capler et al., 2017; Decorte et al., 2017; Feldman & Mandel, 1998; Hathaway & Rossiter, 2007; Lankenau et al., 2018; Valleriani, 2022; Walsh et al., 2013; Pardal, 2016; Parés-Franquero et al., 2019). However, the process of becoming we documented in this study centered low-threshold access because this type of access had been lost post-legalization with the closure of “grey area” cannabis clubs and medical dispensaries. Unlike Athey and colleagues (2017) who conducted their work in the pre-legalization Canadian context, at a time when hundreds of low-threshold access points were operational in British Columbia, the question of access became central to our work and required us to rethink the process of becoming as it had been theorized to date. Therefore, our findings differ in that they describe low-threshold access to community, medicine, and space as a *precursor* to social learning. This is different from the findings of Athey and colleagues (2017), which focused primarily on social learning.

Our findings did not align entirely with Lankenau and colleagues (2018), mainly because California (United States) allows storefront access to medical dispensaries and aspiring medical cannabis users can move from obtaining a medical authorization to accessing medical cannabis and the support of knowledgeable staff in ways that are not currently possible in Canada. However, our findings echoed an important theoretical avenue identified by the authors. Toward the end of their paper, Lankenau and colleagues (2018) mention that their findings point to the “prosocial” and “promedical” role of medical dispensaries (p. 69). In other words, that these dispensaries play an important role in building helpful relationships and directing clients to the right information and the right products in ways that generate benefits for the clients themselves and society more generally. In light of our study, we find this theoretical avenue to be particularly important given the need to revisit Becker's work amid the changing socio-political-legal contexts of medical cannabis. The Canadian context provides a compelling example of why this is needed and how the process of becoming a medical cannabis user can be affected by major policy shifts such as legalization. Being able to articulate how legalization affects this process, for example, is a useful contribution of Becker-informed research.

In light of our study findings, we see three important areas that merit further research. First, we recognize the importance of documenting the experience of medical cannabis users in jurisdictions that undertake cannabis legal reform. This area of research should incorporate an equity and intersectional lens to reach medical cannabis users who have been disproportionately affected by cannabis-related stigma and discrimination, surveillance, reporting, and criminalization as well as those who are socially and structurally vulnerable due to their age, disability, income, social isolation, and housing status. Our study was not designed to evaluate the impact of cannabis legalization in Canada; however, our findings speak to the impact of legalization on low-threshold access to community, medicine, and space and why this matters to medical cannabis users. Second, we see a need for additional research to advance the concept of low-threshold access in the context of medical cannabis. This could include further studies to explore this concept with medical cannabis users who experience access barriers. It could also include studies looking at various models of low-threshold access. Research on the cannabis social club model, for example, offers an interesting starting point for developing and regulating low-threshold access points across socio-political-legal contexts (e.g., legalization, *de facto* decriminalization, *de jure* decriminalization) (Araña & Parés 2020; Belackova & Wilkins, 2018; Decorte, 2015; Decorte et al., 2017; Decorte & Pardal, 2017, 2020; Goumaz et al., 2015; Jansseune et al., 2019; Obradors-Pineda et al., 2021; Pardal, 2016, 2018a, 2018b, 2023; Pardal & Decorte, 2018; Pardal et al., 2022; Parés-Franquero et al., 2019; Queirolo et al., 2016; Zobel et al., 2015). Finally, we believe that more research on the process of becoming a medical cannabis user is needed given that richness and complexity of the experiences documented in this study.

Our theory-informed analysis had some limitations because it was focused on a sub-set of the data consisting of interviews with 12 participants with lived experience who were not representative of medical cannabis users and who were more likely to present a favorable bias toward low-threshold access points. It was also limited to “social learning” data extracted from the interviews. However, to balance these limitations, we conducted a rigorous analysis informed by Becker's original work as well as subsequent publications written by scholars seeking to advance Becker's work through theorizing and studying both recreational and medical cannabis users. We also supported our findings with detailed quotes from as many different participants as possible. To situate our analysis and demonstrate rigor, we provided an overview of the case (i.e., low-threshold access points in British Columbia), explained why this case was instrumental, and how we studied the case by drawing on multiple sources of data (including field work). We believe that our year-long process for building relationships, collecting rich data, organizing the data, and analyzing the data also serves to demonstrate rigor. Overall, we believe that the analysis contributes to an evolving body of literature and addresses important gaps.

## Conclusion

The main contributions of this study are three fold: it offers additional insights for theorizing the process of becoming a medical cannabis user, it introduces the concept of low-threshold access as a way to think through the role and contribution of access points that have historically functioned in the “grey” (outside the medical and legal systems), and it helps to understand the challenges and implications arising from the socio-political-legal context of medical cannabis in Canada. As we have noted elsewhere (Gagnon et al., 2020, 2023), the legalization of cannabis in Canada is an opportunity to make what Watson and colleagues (2019) call “real-time observations” (Watson et al., 2019). This case study allowed us to make real-time observations at a critical juncture in British Columbia. With the closure of all but two low-threshold access points post-legalization, medical cannabis has entered a new era in the province. Lawyer Jack Lloyd describes this era as a “slow eradication of compassionate access to cannabis” (Hager & Imam, 2020). We hope that our study helps to document what this eradication means for medical cannabis users and why low-threshold access is not only compassionate but also effective at promoting and supporting learning, effective medicating, advancing equity, and upholding the rights of medical cannabis users.

## Notes

1. Cannoil is a cannabis infused organic olive oil that can be ingested in oil or capsule format, used to make edibles, or applied to the skin as a topical massage oil, for example.
